# *Helicobacter pylori* Eradication Reverses DNA Damage Response Pathway but Not Senescence in Human Gastric Epithelium

**DOI:** 10.3390/ijms25073888

**Published:** 2024-03-31

**Authors:** Polyxeni Kalisperati, Evangelia Spanou, Ioannis S. Pateras, Konstantinos Evangelou, Irene Thymara, Penelope Korkolopoulou, Athanassios Kotsinas, Panayiotis G. Vlachoyiannopoulos, Athanasios G. Tzioufas, Christos Kanellopoulos, Vassilis G. Gorgoulis, Stavros Sougioultzis

**Affiliations:** 1 Gastroenterology Unit, Department of Pathophysiology, School of Medicine, National and Kapodistrian University of Athens, Mikras Asias 75, 11527 Athens, Greece; evagspa@yahoo.gr; 2 2nd Department of Pathology, “Attikon” University Hospital, Medical School, National and Kapodistrian University of Athens, Rimini 1, 12462 Athens, Greece; ispasath2004@yahoo.com; 3 Molecular Carcinogenesis Group, Department of Histology and Embryology, Faculty of Medicine, National Kapodistrian University of Athens, Mikras Asias 75, 11527 Athens, Greece; cnevagel@med.uoa.gr (K.E.); akotsin@med.uoa.gr (A.K.); vgorg@med.uoa.gr (V.G.G.); 4 1st Department of Pathology, Laiko Hospital, School of Medicine, National and Kapodistrian University of Athens, Mikras Asias 75, 11527 Athens, Greece; ei_thymara@yahoo.gr (I.T.); pkorkol@med.uoa.gr (P.K.); 5 Department of Pathophysiology, School of Medicine, National and Kapodistrian University of Athens, Mikras Asias 75, 11527 Athens, Greece; pvlah@med.uoa.gr (P.G.V.); agtzi@med.uoa.gr (A.G.T.); 6 Faculty of Geology and Geoenvironment, National and Kapodistrian University of Athens, 15771 Athens, Greece; ckanellopoulos@gmail.com; 7 Ninewells Hospital and Medical School, University of Dundee, Dundee DD1 4HN, UK; 8 Division of Cancer Sciences, School of Medical Sciences, Faculty of Biology, Medicine and Health, University of Manchester, Oxford Road, Manchester M13 9PL, UK; 9 Faculty of Health and Medical Sciences, University of Surrey, 30 Priestley Road, Surrey Research Park, Guildford, Surrey GU2 7YH, UK

**Keywords:** *H. pylori*, eradication, DNA damage response, tumorigenesis, intestinal metaplasia, senescence

## Abstract

*Helicobacter pylori (H. pylori)* infection induces DNA Double-Strand Breaks (DSBs) and consequently activates the DNA Damage Response pathway (DDR) and senescence in gastric epithelium. We studied DDR activation and senescence before and after the eradication of the pathogen. Gastric antral and corpus biopsies of 61 patients with *H. pylori* infection, prior to and after eradication treatment, were analyzed by means of immunohistochemistry/immunofluorescence for DDR marker (γH2AΧ, phosporylated ataxia telangiectasia-mutated (pATM), p53-binding protein (53BP1) and p53) expression. Samples were also evaluated for Ki67 (proliferation index), cleaved caspase-3 (apoptotic index) and GL13 staining (cellular senescence). Ten *H. pylori* (−) dyspeptic patients served as controls. All patients were re-endoscoped in 72-1361 days (mean value 434 days), and tissue samples were processed in the same manner. The eradication of the microorganism, in human gastric mucosa, downregulates γH2AΧ expression in both the antrum and corpus (*p* = 0.00019 and *p* = 0.00081 respectively). The expression of pATM, p53 and 53BP1 is also reduced after eradication. Proliferation and apoptotic indices were reduced, albeit not significantly, after pathogen clearance. Moreover, cellular senescence is increased in *H. pylori*-infected mucosa and remains unaffected after eradication. Interestingly, senescence was statistically increased in areas of intestinal metaplasia (IM) compared with adjacent non-metaplastic mucosa (*p* < 0.001). In conclusion, *H. pylori* infection triggers DSBs, DDR and senescence in the gastric epithelium. Pathogen eradication reverses the DDR activation but not senescence. Increased senescent cells may favor IM persistence, thus potentially contributing to gastric carcinogenesis.

## 1. Introduction

*Helicobacter pylori* is a Gram-negative bacterium that colonizes the gastric mucosa of almost 50% of the world population, resulting in lifelong infection and gastritis [1,2]. The great majority of the infected hosts remains asymptomatic. However, under certain conditions related to the pathogen’s virulence factors, host susceptibility and genetics, and environmental cofactors, *H. pylori* infection may result in peptic ulcer, lymphoma of the Mucosa-Associated Lymphoid Tissue and gastric cancer [2,3,4,5]. *H. pylori* predisposes humans to gastric cancer, and this association has been confirmed epidemiologically, although the mechanistic details at the histological and molecular levels have not yet been clarified. Nevertheless, *H. pylori* has been classified as a human carcinogen class I [6,7,8,9]. The well-accepted Correa model describes the progression of *H. pylori* gastritis to gastric cancer through the preneoplastic lesions of atrophy, intestinal metaplasia (IM) and dysplasia [10].

Persistent inflammation has been associated with carcinogenesis through the promotion of continuous injury and repair, which induces aberrant cell proliferation and subsequent replication stress that favors the formation of DNA lesions [11]. Among them, Double-Strand Breaks (DSBs) pose the greatest challenge for genome integrity and cell fate, since if left unrepaired they are lethal, while error prone repair underlies genetic instability and the emergence of pathological conditions, such as malignancies [12,13]. DSBs occurrence results in DNA Damage Response (DDR) pathway activation in order to signal the lesions’ presence and repair [14,15]. Specifically, DSBs promote ataxia telangiectasia-mutated (ATM) and ATM-Rad3-related (ATR) activation, resulting inH2AX histone phosphorylation at Ser 139 (γH2AΧ) [16,17,18]. γH2AΧ, in turn, activates the transducer factorsChk1 and Chk2, which activate p53 [17,19,20]. p53 activation halts cell cycle progression, allowing the cell either to repair, in an error-free manner, the lesion or induce the anti-tumor barriers of apoptosis or senescence to prevent malignant progression upon error-prone DNA repair. Apoptosis is a type of cell death and considered to be the cellular response to overwhelming stress, whereas senescence ensues when damage is less severe [21]. Senescent cells are generally in an irreversible cell cycle arrest condition, non-proliferative but viable and metabolically active as they secrete various factors modulating the extracellular environment and neighboring cells, termed SASPs (senescence associated secretory phenotypes) [22,23,24,25]. Both apoptosis and senescence contribute to tissue homeostasis and health by either eliminating or preventing the propagation of damaged cells, respectively [26]. However, DNA replication stress promotes DSBs formation, leading to genomic instability and selective pressure for p53 mutations, abrogating the tumor-suppressing actions of p53. When the DDR pathway experiences inefficiency due to overburden or p53 mutations, faithful DNA repair is compromised, which ultimately leads to genomic instability.

Previous studies have linked *H. pylori* infection to DSB formation and DDR activation [27,28,29,30,31,32,33]. The available data suggest that *H. pylori* infection promotes the formation of DSBs either directly through host–pathogen contact or indirectly via the chronic inflammation that accompanies the infection; both in vitro and in vivo evidence indicates that this ultimately leads to DDR activation. The DDR pathway may be compromised by persistent infection and inflammation, which may result in p53 mutations, unrepaired DNA damage, and even tumor development. In the current study, we investigated DDR activation via the immunohistochemical/immunofluorescent assessment of γH2AΧ, pATM, 53BP1 and p53 expression in gastric biopsies of *H. pylori*-infected patients before and after eradication treatment. Moreover, we evaluated cellular senescence in *H. pylori* (+) patients and examined the impact of *H. pylori* clearance both on DDR and senescence. Finally, we studied cellular senescence in premalignant lesions, especially in *H. pylori*-related intestinal metaplasia.

## 2. Results

### 2.1. H. pylori Infection Activates DDR

**Figure 1:**
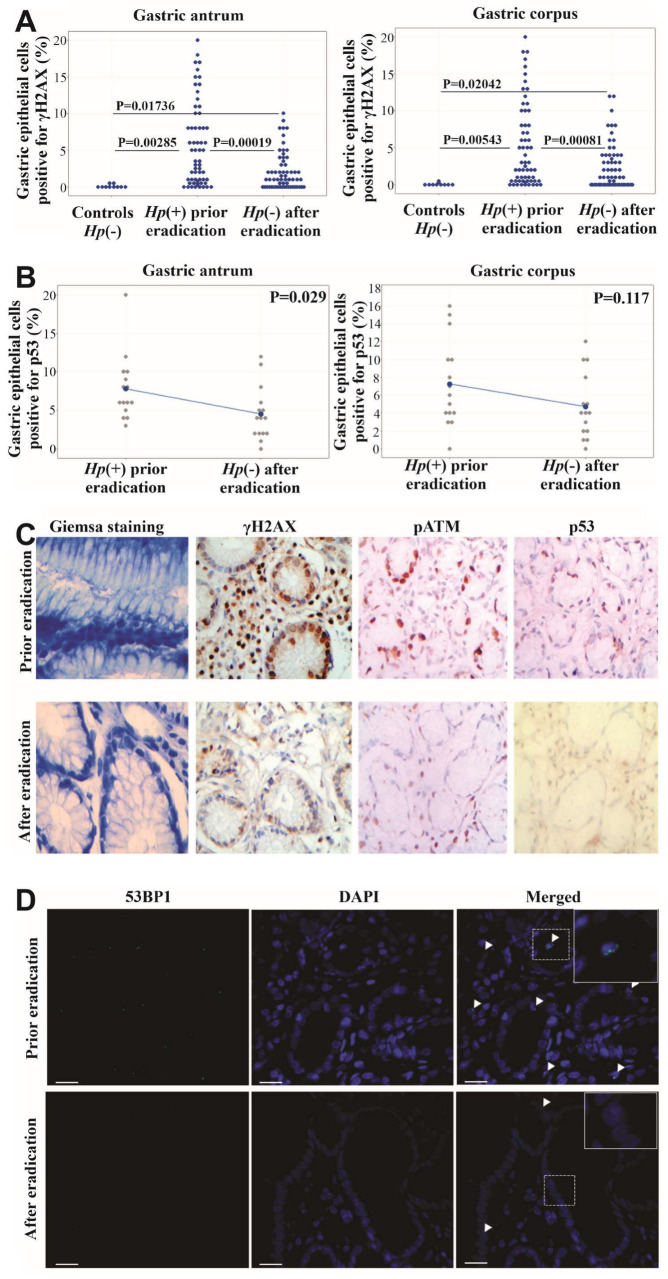
DDR marker activation assessment in gastric biopsies. (**A**) γH2AΧ (N = 61 samples of *H. pylori* (+) examined) expression prior to and after eradication of *H. pylori* in gastric antrum and corpus compared to *H. pylori* (−) controls (N = 10). *p* < 0.01667 (adjusted Bonferroni significance) between *H. pylori* (+) and *H. pylori* (−) patients, as well as prior to and after eradication. (**B**) p53 (N = 15 samples examined) expression prior to and after eradication of *H. pylori* in gastric antrum and corpus. *p* < 0.05 (statistically significant) prior to and after eradication in antrum. (**C**) γH2AΧ, pATM and p53 immunohistochemical expression, before and after eradication of *H. pylori* in same patient. Giemsa stain (1st column) is included to confirm the infection status. Note: significant decrease after eradication. γH2AΧprior 10% vs. γH2AΧafter 2%, pATMprior 11% vs. pATMafter 3% and p53prior 3% vs. p53after 1%. (**D**) Decreased formation of 53BP1foci after eradication of *H. pylori*. 53BP1 foci prior to eradication 7% vs. 53BP1 foci after eradication 2%. Inserts depict areas of higher magnification. Scale bar: 50 μm.

γH2AΧ immunohistochemical activity was significantly increased in the gastric epithelial cells of 61 *H. pylori*-infected patients compared with 10 *H. pylori* (−) controls, both in the antrum and corpus of the stomach, indicating DDR activation (adjusted Bonferroni *p* = 0.00285 and *p* = 0.00543, respectively) (Figure 1A). We studied only 10 *H. pylori* (−) patients since we observed striking differences in γH2AX immunohistochemical expression compared to *H. pylori* (+) patients, in keeping with data previously published by Xie et al. [30]. pATM and p53 immunohistochemical expression were also increased in 15 *H. pylori* (+) patients, confirming DDR activation. 53BP1 foci formation via immunofluorescence was also found increased in randomly selected *H. pylori* (+) patients. γH2AΧ expression was not associated with patients’ ages (>60 y.o. vs. <60 y.o., *p* = 0.755) or sex (*p* = 0.979) or the endoscopic findings (peptic ulcer vs. no peptic ulcer, *p* = 0.801). In addition, γH2AΧexpression, in *H. pylori* (+) patients, was unaffected by the presence or absence of IM in the gastric epithelium (*p* = 0.82). For atrophy, γH2AΧ expression was found slightly lower in *H. pylori* (+) patients without atrophy vs. *H. pylori* (+) patients with atrophy (*p* = 0.196). 

### 2.2. H. pylori Eradication Reduces DDR Activation

A statistically significant decrease in γH2AΧ expression was observed after *H. pylori* eradication both in the antrum (mean_prior_ = 6.03 vs. mean_after_ = 3.12; SEM_prior_ = 0.51 vs. SEM_after_ = 0.31) and corpus (mean_prior_ = 5.15 vs. mean_after_ = 2.41; SEM_prior_ = 0.93 vs. SEM_after_ = 0.53) of 61 included patients (adjusted Bonferroni *p* = 0.00019 and *p* = 0.00081, respectively) (Figure 1A). p53 immunohistochemical analysis in 15 randomly selected cases also revealed decreased expression after eradication treatment that was statistically significant in the gastric antrum (*p* = 0.029) but not in corpus (*p* = 0.117) (Figure 1B). In addition, both pATM and 53BP1 foci formation were decreased after eradication in randomly selected cases (Figure 1C,D). γH2AΧ expression decline seems to correlate with time. Indeed, we observed a trend toward the less expression of γH2AΧ when repeat endoscopy was performed in a period > 1 year vs. period < 1 year after the initial one (*p* = 0.24).

### 2.3. Epithelial Cell Kinetics in H. pylori-Infected Patients

The cellular proliferation of gastric epithelial cells, as studied via immunohistochemical analysis of the Ki67 status, revealed a non-significant decrease after *H. pylori* eradication in both the gastric antrum (mean_prior_ = 22.3 vs. mean_after_ = 19.6; SEM_prior_ = 2.7 vs. SEM_after_ = 3) and corpus (mean_prior_ = 20.27 vs. mean_after_ = 14.33; SEM_prior_ = 2.2 vs. SEM_after_ = 2.1) (*p* = 0.499 and *p* = 0.066 respectively). Cellular apoptosis was also studied via the immunohistochemical analysis of cleaved caspase 3, which also revealed no statistically significant difference before and after eradication treatment in both the antrum and corpus (*p* = 0.725 and *p* = 0.672 respectively).

### 2.4. Cellular Senescence Is Increased in H. pylori (+) Gastric Biopsies

**Figure 2:**
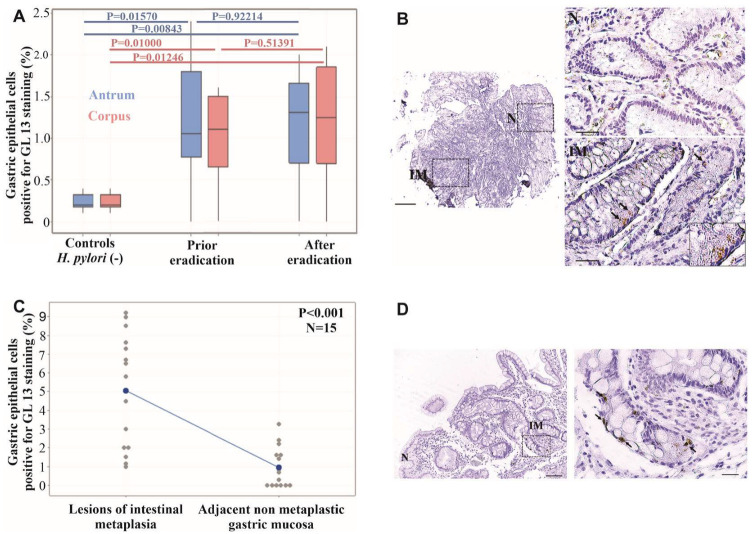
Evaluation of senescence in *H. pylori* gastritis. (**A**) GL13 immunohistochemical expression is increased in *H. pylori* (+)-infected gastric mucosa (N = 10) vs. the *H. pylori* (-) (N = 10) (*p* = 0.01570 and *p* = 0.01000 for the gastric antrum and corpus, respectively) and remains unaffected by the eradication of the pathogen in both the antrum and corpus (adjusted Bonferroni significance *p* < 0.01667). (**B**) Increased expression of GL13 in areas of intestinal metaplasia (IM) (lower insert) vs. the adjacent non-metaplastic mucosa (N) (upper insert) in gastric specimens of *H. pylori*-infected patients. (**C**) Statistically significant increase in SenTraGor^TM^ (GL13) expression via hybrid immunohistochemical analysis in areas of intestinal metaplasia vs. the adjacent non-metaplastic mucosa in *H. pylori* infection (N = 15). (**D**) Increased expression of p16^INK4A^ via immunohistochemical analysis in areas of intestinal metaplasia vs. adjacent non-metaplastic mucosa in a *H. pylori*-infected patient. Scale bars: 200 μm (lower magnification) and 50 μm (higher magnification).

Cellular senescence was examined using hybrid immunohistochemical analysis with SenTraGor^TM^ (GL13) in 10 *H. pylori* (+) patients and 10 *H. pylori* (−) patients. Statistical analysis showed that cellular senescence was significantly increased in *H. pylori* (+) gastric epithelium vs. *H. pylori* (−)in both the antrum and corpus (adjusted Bonferroni *p* = 0.01570 and *p* = 0.01000, respectively) (Figure 2A).

### 2.5. Cellular Senescence Is not Reversed via H. pylori Eradication

We examined cellular senescence in gastric biopsies of 10 *H. pylori* (mean time between endoscopies = 377 days)-infected patients before and after eradication treatment via hybrid immunohistochemical analysis with SenTraGor^TM^ (GL13). Statistical analysis revealed that cellular senescence was unaffected by pathogen eradication in both the antrum and corpus (adjusted Bonferroni *p* = 0.92214 and *p* = 0.051391, respectively) (Figure 2A).

### 2.6. Increased Cellular Senescence in Lesions of Intestinal Metaplasia of H. pylori-Infected Patients

Hybrid immunohistochemical analysis with SenTraGor^TM^ was performed in order to study cellular senescence in areas of IM in *H. pylori* (+) patients and showed increased levels of SenTraGor^TM^ (GL13) in lesions of IM vs. the adjacent gastric mucosa without lesions of IM (*p* < 0.001) (Figure 2B,C). Increased senescence in areas of intestinal metaplasia was also confirmed via elevated immunohistochemical staining for p16^INK4A^ in randomly selected cases (Figure 2D).

## 3. Discussion

In the present study, we provide evidence that *H. pylori* upregulates DDR and induces senescence in gastric epithelial cells. The eradication of the microorganism reverses DDR activation but not senescence. Interestingly, senescent cells were more abundant in areas of IM.

DDR activation was confirmed by the aforementioned markers, namely γH2AΧ, pATM and p53 status, along with 53BP1 foci. γH2AΧ expression was not associated with patients’ age or sex, endoscopic findings or the presence of the premalignant lesions of atrophy and IM. Taking into consideration that atrophy and IM indicate chronicity, we suggest that DSB formation and DDR activation are early events in the course of *H. pylori* infection. It should be noted that Xie et al. also studied γH2AΧ expression in human gastric specimens and observed a gradual, statistically significant, increase in the histological lesions of chronic gastritis, IM to dysplasia. In the same study, γH2AΧ expression in the above-mentioned lesions was higher in *H. pylori* (+) patients vs. *H. pylori* (−) patients [30]. Moreover, Krishnan et al. demonstrated γH2AΧ expression in *H. pylori*-infected human gastric biopsies and observed a gradual increase in γH2AΧ through chronic gastritis and IM, but the findings were not statistically significant [31].

In our study, DDR activation was reduced after *H. pylori* eradication, as assessed via γH2AΧ, pATM and p53 immunohistochemical staining, both in the gastric antrum and body. This reduction was greater in the gastric antrum, as indicated by the statistical analyses of γH2AΧ and p53 immunohistochemical expression (*p* = 0.00019 and *p* = 0.029, respectively). The greater reduction in DDR in the antrum may be explained by *H. pylori’s* tropism for gastric antrum and the increased population of the bacterium in this compartment. The reduction in p53 expression is in accordance with Satoh et al., who previously reported a decline in p53 expression after eradication treatment [34].

Taken together, the above data support the idea that *H. pylori* infection induces DSBs in gastric epithelium, leading to the activation of DDR in epithelial cells, a process that seems to reverse after eradication. Broadly speaking, DDR and the resultant p53 activation may lead to epithelial cell apoptosis or senescence that serves as anti-tumor barriers.

**Figure 3:**
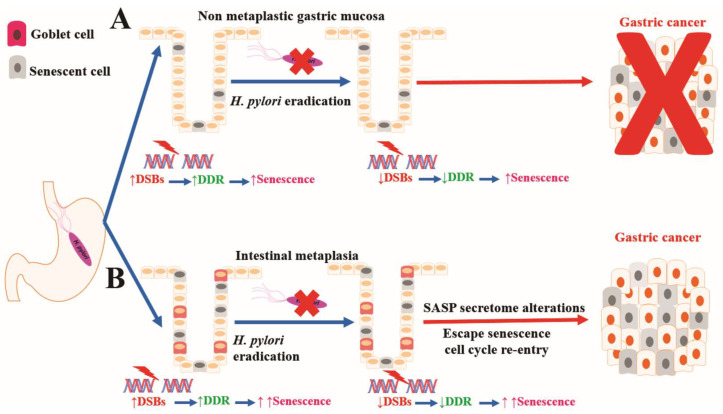
Proposed role of senescence in *H. pylori* gastritis. *H. pylori* induces DSB formation, DDR activation and senescence in the gastric epithelium. (**A**) In the absence of premalignant lesions, senescent cells, which persist after eradication, serve as an effective tumor barrier, preventing gastric cancer development. (**B**) In areas of IM, senescent cells are increased in number, probably due to enhanced stressful stimuli, and their number is unaffected after eradication. We suggest that under the influence of various factors, such as p53 mutations due to continuous replication stress in the hyperproliferative IM, senescent cells may alter their SASP secretomes or re-enter the cell cycle, acquiring aggressive behavior, eventually favoring tumorigenesis.

Cellular senescence was increased in *H. pylori* (+) gastric epithelium vs. *H. pylori* (−) controls in both the gastric antrum and corpus (adjusted Bonferroni *p* = 0.01570 and *p* = 0.01246, respectively). Senescence evaluation was performed by applying a novel and reliable method of hybrid immunohistochemistry with GL13 (SenTraGor^TM^), a compound that was developed by our laboratory and detects lipofuscin, a by-product of the senescence process and “hallmark” of senescent cells. Indeed, lipofuscin is a nondegradable aggregate of oxidized lipids, amino acids, oligosaccharides and metals that accumulates in lysosomes of aged postmitotic cells [35,36]. Interestingly, the number of senescent cells was found to be unaffected after *H. pylori* eradication, an observation that was consistent irrespective of the time interval between the initial and repeat endoscopies (mean of 377 days, range 85–1461 days). Our findings are in accordance with Cai et al., who reported increased senescence in *H. pylori*-infected atrophic mucosa as a result of NF-κB driven CXCR2 upregulation [37]. The authors speculate that senescence leads to atrophy development and *H. pylori* is dispensable once abundant senescence is activated, possibly due to increased CXCR2 expression by epithelial cells or/and IL-1^α^ from SASPs. However, no follow-up evaluation was designed, as was performed in our study, to document the persistence of senescent cells after *H. pylori* eradication. The persistence of senescent cells in gastric epithelium after *H. pylori* eradication, especially in areas of IM (Figure 2A), may arrest the progression of metaplastic epithelium to more advanced lesions such as dysplasia and gastric cancer, which is in keeping with the main physiological role of senescence. However, it should be mentioned that senescence may act either as an anti-tumorigenic factor or oncogenic stimulant [22,38]. Consequently, during IM progression, various factors, i.e., p53 mutations, may alter SASP secretomes and abolish the protective role of senescence. Indeed, SASP-secreted factors promote tumor progression in high-grade preneoplastic lesions and cancer [38]. Moreover, the increased expression of p16 ^INK4A^ in areas of IM (Figure 2D) may aid the reprogramming of epithelial cells, i.e., chief cells, of the gastric gland that drives the development of IM or contributes to its maintenance [39,40,41]. On the other hand, recent interesting research has provided evidence and mechanistic details supporting the idea that senescent cells may re-enter cell cycle and exhibit aggressive behavior [42]. Taken together, all the above findings suggest that senescent cells in areas of IM may fuel gastric tumorigenesis, potentially explaining the loss of the benefit of *H. pylori* eradication in gastric cancer prevention in cases in which precancerous lesions, such as IM and dysplasia, have developed [43,44,45] (Figure 3). IM is considered to be “a point of no return”, meaning that it is not reversible after H. pylori clearance since it frequently contains p53 mutations. Therefore, the European Society of Gastrointestinal Endoscopy (ESGE) and the American Gastroenterological Association (AGA) suggest performing endoscopic surveillance in patients with histologically confirmed IM [46,47].

The analysis of proliferation and apoptosis indices of gastric epithelial cells revealed no significant difference before and after eradication treatment. Of note, the proliferation index (Ki67) is reduced after eradication treatment, but without statistical significance. These results confirm previously reported data from our group and others showing residual proliferative activity after eradication [48,49]. For the apoptotic index, an increase in *H. pylori*-infected mucosa compared to *H. pylori* (−) controls was observed that was not significantly reduced after eradication, in contrast to data previously published by us and others [49,50,51,52]. It should be noted that these studies used a TUNEL assay for apoptosis estimation, which is considered to be a more sensitive method than the cleaved caspase3 used in the present study [53]. Interestingly, it has been reported that senescent cells, via their secretome profile, may promote the proliferation of adjacent cells, thus providing a possible explanation for the observed residual hyperproliferation of gastric epithelial cells after *H. pylori* eradication [23,54,55]. Moreover, the increased number of senescent cells in areas of IM may confer the acknowledged hyperproliferative capacity of the metaplastic epithelium [56,57].

## 4. Patients and Methods

### 4.1. Patients

Data were retrospectively collected in the Gastroenterology outpatient Department of the Pathophysiology Clinic from unselected dyspeptic patients who attended for upper gastrointestinal endoscopy between January 2015 and January 2019. Biopsy samples, after pathological diagnosis, were collected and stored in the Pathology Department and processed as archival specimens. Patients were included if (1) they were tested *H.pylori* (+), as confirmed via both a rapid urease test (Campylobacter-Like Organisms (CLO) test) and histological identification of the pathogen (Giemsa stain); (2) they were above 18 years old; and (3) they provided informed consent to be subjected to upper endoscopy. Patients on antisecretory treatment including Proton-Pump Inhibitors (PPI’s) or H_2_ receptor antagonists, patients who reported previous eradication treatment for *H. pylori* or gastric surgery, patients on NSAIDS or aspirin treatment, pregnant women and the critically ill were excluded. In total, 61 *H. pylori* (+) patients who attended for a repeat endoscopy upon clinical indications, mainly symptom persistence or reappearance, after the eradication of the pathogen were enrolled as a study group. Ten dyspeptic patients meeting the above criteria and tested negative for *H. pylori* via both the above-mentioned methods served as controls. This study was performed according to the Declaration of Helsinki and approved by the Ethics Committee of “Laiko” General Hospital of Athens.

### 4.2. Study Design

All patients included in the present study underwent upper gastrointestinal endoscopy, and gastric biopsies were taken according to the Sydney protocol (2 from the antrum, 2 from the corpus and 1 from the incisura angularis) [58]. All patients were also tested via a CLO test for *H. pylori*. Patients found to be positive for *H. pylori* infection via both methods were treated with an eradication treatment consisting of esomeprazole 40 mg twice a day (bis in die, bid), amoxicillin 1 gram bid, clarithromycin 500 mg bid and metronidazole 500 mg bid for 10 days. Patients with peptic ulcer were prescribed esomeprazole 40 mg bid for an additional 3 weeks after the eradication treatment. A second endoscopy was performed after the initial one, at least two months after the completion of the eradication regimen. The eradication of the pathogen was confirmed via both histology (Giemsa stain) and an Urea Breath Test. The mean time between the two endoscopies was 434 days. In total, 42/61 patients had an endoscopic image of predominant antral gastritis, 4/61 had one for corpus gastritis, 4/61 had one for duodenal ulcer and 11/62 had one for gastric ulcer. The men to women ratio in the examined cohort was 1.2:1. The characteristics of the study group are reported in Table 1.

### 4.3. Histopathology

Formalin-fixed, paraffin-embedded tissue samples were cut at 4 μm and stained with Giemsa and hematoxylin-eosin. Two expert pathologists (VG, PK) independently reviewed the gastric specimens for chronic inflammation, activity, atrophy, intestinal metaplasia and *H. pylori* density, according to the criteria of the updated Sydney system [58].

### 4.4. Immunohistochemical and Immunofluorescent Analysis

For immunohistochemical and immunofluorescent analysis, previously described protocols were applied using the following antibodies: anti-phospho H2AX [(Ser 139, clone JBW301, 06-636, Millipore, Merck, MA, USA) (mouse monoclonal), 1:1000], anti-p53 [(clone DO7, sc47698, Santa Cruz, TX, USA), (mouse monoclonal), 1:100], anti-phospho ATM [(Ser 1981, 10H11.E12, Merck, MA, USA) (mouse monoclonal), 1:1000], anti-Ki67 [(ab16667, Abcam, MA, USA), 1:100], anti-cleaved caspase 3 [(9661L, Cell Signaling, MA, USA), 1:200], anti-p16^INK4A^ [(clone:E6H4, Roche, Rotkreuz, Switzerland), (mouse monoclonal), ready to use], and anti-53BP1 [(ab36823, Abcam, MA, USA), (rabbit polyclonal), 1:250] [42,59,60]. Regarding immunohistochemistry, all steps including tissue section deparaffinization, rehydration, antigen retrieval, endogenous peroxidase activity inactivation, primary and secondary antibody incubation, followed by diaminobenzidine substrate-based signal development, have been previously described [42,60,61,62]. For immunofluorescent analysis, tissue sections were processed, as previously reported [42,60], and observed using a Zeiss Axiolab fluorescence microscope (Fisher scientific, Leicestershire, UK) equipped with a Zeiss AxiocamMRm camera and Achroplan objectives, while image acquisition was performed with AxioVision software ver. 4.7.1.

### 4.5. Review and Scoring

Sections subjected to immunohistochemistry were reviewed and scored independently by two experienced pathologists and evaluated for staining frequency. Specifically, an average of 500 cells was counted at x400 magnification for each case, with minimal inter-observer variability (*p* < 0.001). For γH2AX, pATM, p53, Ki67 and cleaved caspase 3 evaluation, the number of positively stained nuclei was expressed as the percentage of positive nuclei to the total number of counted nuclei/HPF. For immunofluorescence, sections were also reviewed and scored by two experienced pathologists. Nuclei stainings for 53BP1 foci were scored as positive, and the percentage of the positive nuclei was calculated.

### 4.6. Senescence–SenTraGor^TM^ (GL13) Staining and Assessment

For senescence evaluation, a hybrid immunohistochemistry was performed, as previously described, and recommended using the SenTraGor^TM^ (trademark of GL13) compound [35,61,63]. A secondary anti-biotin [(dilution Hyb-8, ab201341, Abcam) 1:300] antibody was used against the biotin moiety of the SenTraGor^TM^ (GL13) compound. Signal development was as per the imunohistochemical analysis described above. GL13 is a biotinylated Sudan Black-B (SBB) chemical analog that interacts potently and specifically with lipofuscin, a molecule that is inherently linked to the senescence process. The method has been reported to outperform most of the available senescent cell-staining methods with respect to their technical challenges, limitations and errors [35,36]. The mean percentage of SenTraGor^TM^ (GL13)-positive gastric epithelial cells in at least 10 high power fields (x400) per patient was quantified. In cases of IM, senescence was evaluated as the mean percentage of SenTraGor^TM^ (GL13)-positive gastric epithelial cells separately in metaplastic and non-metaplastic glands in the same tissue sections (Figure 2B,C).

### 4.7. Statistical Analysis

Data were expressed as the mean and standard error of the mean (SEM). Two sample *t* tests were applied to compare values between two groups of data, using Minitab statistical software ver. 19 (Minitab, Coventry, UK). *p* < 0.05 was considered to indicate statistical significance. One-way Anova with Bonferroni correction was applied to compare values between three groups of data, using Microsoft Excel 365 software. *p* < 0.01667 was considered to indicate statistical significance.

## 5. Conclusions

In conclusion, *H. pylori* induces DSBs in gastric epithelial cells that probably contribute to the cascade of gastric carcinogenesis through the known precursor lesions of atrophy, IM and dysplasia. The consequent DDR activation and senescence mainly act as anti-tumor barriers, further reinforced by our finding of increased senescent cells in areas of IM. However, it is tempting to speculate that under the influence of various factors such as p53 mutations, senescent cells may alter SASP secretome or re-enter the cell cycle, potentially favoring tumorigenesis (Figure 3). Future research will hopefully shed more light on the mechanistic details of the interplay between *H. pylori*, IM, senescence and gastric cancer development.

## Figures and Tables

**Table 1:** Characteristics of the study group.

Characteristics	Values	
**Sex**	Men	34
	Women	27
**Time interval between the two endoscopies**	72–1361 days	434 days
**Endoscopic findings**	Antral gastritis	42/61 (69%)
	Corpus gastritis	4/61 (6.5%)
	Duodenal ulcer	4/61 (6.5%)
	Gastric ulcer	11/61 (18%)
**Age**	28–82 years old	58 years old
**Histological findings**	Chronic active gastritis	55/61 (90%)
	Atrophy	58/61 (95%)
	Intestinal metaplasia	25/61 (41%)

## Data Availability

Data available on reasonable request.
